# The homologous tumor‐derived‐exosomes loaded with miR‐1270 selectively enhanced the suppression effect for colorectal cancer cells

**DOI:** 10.1002/cam4.6936

**Published:** 2024-01-10

**Authors:** Yingmin Jin, Liying Sun, Yingying Chen, Yue Lu

**Affiliations:** ^1^ Department of Gastroenterology The First Affiliated Hospital of Harbin Medical University Harbin China

**Keywords:** colorectal cancer, efficacy evaluation, engineered exosomes, safety evaluation

## Abstract

**Background:**

Colorectal cancer (CRC), known as prevalent cancer, has risen to be the leading cause of cancer‐related death. Engineered exosomes had attracted much attention since they acted as carriers to deliver small molecule drugs, therapeutic nucleic acids, and polypeptides to treat a series of cancers.

**Methods and Results:**

Here, we found that the PKH‐26 labeled exosomes, which were derived from the CRC cells, could be efficiently absorbed by SW1116 cells and had an abundant fluorescence distribution in tumors, compared with the exosomes derived from mesenchymal stem cells (MSC) and HepG2 cells. This Research demonstrated that engineered CRC‐exosomes loaded with functional miR‐1270 (Exo‐miR‐1270) enriched in miR‐1270 strongly inhibited the proliferation by CCK‐8 and EdU assays, migration by wound‐healing and transwell assays, and promoted the apoptosis for CRC cells through flow cytometry. MiR‐1270 overexpression delivered by CRC exosomes contributed to inhibiting the tumor growth potential of CRC in vivo and increasing the overall survival of the mice. Moreover, the safety evaluation results showed that CRC‐exosomes loaded with functional miR‐1270‐mimics had no toxicity for other organs by histopathological analysis and no influence on the vital chemistry and hematology parameters for mice in vivo safety evaluation.

**Conclusion:**

These results indicate that Exo‐miR‐1270 can effectively treat CRC tumors by intravenous administration. Our work provided a foundation that the homologous tumor‐derived exosomes mediated miRNA delivery for the treatment of CRC.

## Abstract 2

This study demonstrated that Exo‐miR‐1270 can effectively treat CRC tumors by intravenous administration. Our work lays the foundation for homologous tumor‐derived exosomes‐mediated miRNA delivery for the treatment of CRC.

## 1 INTRODUCTION

Colorectal cancer (CRC) has high morbidity and mortality worldwide, accompanied by high healthcare costs.^1^ According to global cancer statistics in 2018, CRC accounted for approximately 10.2% of total diagnosed cancer cases.^2^ In recent years, with the widespread application of endoscopic and surgical local excision, the clinical treatment of CRC has made significant progress and improved the overall survival of patients. However, CRC is still ranked the fourth leading cause of cancer deaths.^3^

MicroRNAs (miRNAs) are ~20 *nt* small non‐coding RNAs and have significant regulatory functions in CRC.^4^, ^5^, ^6^ MiR‐1270 was one of the widely studied miRNAs reported as an oncogene or an anti‐oncogene in various human diseases and cancers. For instance, miR‐1270 showed an aberrant upregulation in papillary thyroid cancer patients and cell lines.^7^ In addition, miR‐1270 played an oncogene role in osteosarcoma^8^ and non‐small cell lung cancer.^9^ However, miR‐1270 had a typical expression pattern through WT1 in glioblastoma cancer cell lines and clinical samples.^10^ Furthermore, it showed an anti‐oncogene function in hepatocellular carcinoma by targeting AFP, PLAG1 Like Zinc Finger 2, or CENPM.^11^, ^12^, ^13^ Studies have shown that miR‐370‐5p and miR‐140‐5p, as anticancer genes of CRC, are significantly low expressed in CRC tissues and cells, and participate in regulating the development of rectal cancer.^14^, ^15^ MiR‐1270 plays an important role in regulating the proliferation and migration of gastric cancer cells^16^ and hepatocellular carcinoma cells.^17^ However, the role and molecular mechanism of miR‐1270 in CRC have not been reported.

The exosomes (Exo) are membrane vesicles with a diameter of 30–150 nm.^18^ They could be delivered to the recipient cells and microenvironment to participate in a series of biological processes and mediate the cancer progression.^19^, ^20^ So far, exosomes mainly come from stem cells, dendritic cells, and cancer cells. As the exosome source, mesenchymal stem cells (MSCs) were reported to possess distinct advantages, including higher release amounts of the exosome and more stability and sustainability.^21^ The exosomes secreted from HepG2 cells regulated the development of many cancers and diseases.^22^, ^23^ The engineered exosomes derived from SW1116 cells had a high homology for the treatment of CRC. Compared with traditional synthetic delivery vectors, homologous exosomes have many advantages, such as limited immunogenicity, cell targeting, stronger circulatory stability, and biocompatibility. In addition, CD47 in the exosomes plays an important role in avoiding phagocytosis and increasing accumulation in tumor tissues.^24^ Engineered exosomes, as effective and stable carriers, had appeared and aroused great attention. It was reported to deliver functional nucleic acid, polypeptide, and molecule drugs to particular tumor cells, which expanded the opportunities to develop novel and practical approaches for targeted cancer therapy.^25^, ^26^, ^27^, ^28^

Most therapeutic applications of miRNA required packaging the nucleic acid in a vector, including the exosomes, liposomes, and polycationic polymers.^29^, ^30^, ^31^ However, the potential functions for engineered exosomes loaded with therapeutic miRNA were little reported to treat CRC. In this work, the engineered CRC‐exosomes loaded with miR‐1270 were prepared. Moreover, we investigated its roles in CRC cells, observed the distribution in mice, and evaluated its efficacy and safety. Thus, our work put forward a new perspective on CRC treatment.

## 2 MATERIALS AND METHODS

### 2.1 Cell purchase and culture

MSC (species: human), HepG2 (human liver cancer cells), and SW1116 cells (human colorectal adenocarcinoma cells) were purchased from the Chinese Academy of Science cell bank. MSC cells were cultivated in Mesenchymal Stem Cell Basal Medium (6114011, Dakewe, Beijing, China), HepG2 cells were grown in DMEM (11995065, Gibco, CA, USA), and SW1116 cells were maintained in Leibovitz's L‐15 (11415114, Gibco, CA, USA). All cultures were supplemented with 10% FBS (10100147C, Gibco, CA, USA) in constant temperature incubator under the condition of 37°C, 5% CO_2_ for culture.

### 2.2 The preparation and transmission electron microscopy identification for the engineered exosome

#### 2.2.1 Exosome isolation

The exosomes from the MSC (MSC‐Exo), HepG2 (HCC‐Exo), and SW1116 (CRC‐Exo) cell supernatant were isolated through the density gradient ultracentrifugation method according to relevant literatures.^32^, ^33^ Briefly, 2 × 10^7^ cells were cultured in 10 mL DMEM with 10% exosome‐free FBS (System Biosciences, Palo Alto, CA, USA). After culturing for 48 h until the cell reached 80% confluence, 210 mL medium was collected into 50 mL. Centrifuge cells at 4 °C, 300 g for 10 min, 2000 g for 10 min, and then 10,000 g for 30 min to remove adherent cells, dead cells, and cell debris. Then, the supernatant was removed to a new 50 mL tube and centrifuged at 4 °C, 100000 g for 70 min to collect exosomes. Next, the supernatant was carefully removed, and the pellet was re‐suspended with 35 mL PBS to wash the exosomes. Finally, the sample was centrifuged for another 70 min at 4 °C, 100000 g, and the pellet was re‐suspended with 1 × PBS (50 μL) and stored at −80°C.

#### 2.2.2 TEM

For the morphology, the identification of the exosomes was performed by TEM. Specifically, the exosome pellets were fixed by 2.5% glutaraldehyde, dehydrated with increasing alcohol concentrations, and observed under transmission electron microscopy (TEM) (JEM‐1400Flash, JEOL, Tokyo, Japan).

#### 2.2.3 Exosomes loading

MiR‐370‐5p, miR‐1270 or miR‐140‐5p mimics (400 nM) were loaded into the prepared exosomes (10 μg) by electroporation as previously described respectively.^27^ The mixture of miRNA and exosomes were electroporated at 400 V, 50 μF, 30 ms pulse per 2 s pause for three cycles. MiR‐370‐5p mimics (sense: 5′‐CAGGUCACGUCUCUGCAGUUAC‐3′; antisense: 5′‐GUAACUGCAGAGACGUGACCUG‐3′); miR‐1270 mimics (sense: 5′‐CUGGAGAUAUGGAAGAGCUGUGU‐3′; antisense: 5′‐ACACAGCUCUUCCAUAUCUCCAG‐3′); miR‐140‐5p mimics (sense: 5′‐CAGUGGUUUUACCCUAUGGUAG‐3′; antisense: 5′‐CUACCAUAGGGUAAAACCACUG‐3′). After electroporation, exosomes were washed two times in PBS with 100,000 × g centrifugation for 70 min to remove free miR‐370‐5p, miR‐1270, or miR‐140‐5p mimics and unloaded exosomes.

### 2.3 Western blot

The protein samples of the cells and exosomes were collected, lysed in RIPA buffer (high) (R0010, Solarbio, Shanghai, China) on ice for 20 min, and quantified by BCA protein assay kit (PC0020, Solarbio, Shanghai, China). In our work, all antibodies were purchased from Abcam (Shanghai, China) and Proteintech (Wuhan, China). TSG101 (ab125011), CD63 (ab134045), Grp94 (ab238126), MMP‐2 (ab181286), MMP‐9 (ab137867), N‐cadherin (22018‐1‐AP), and vimentin (10366‐1‐AP) antibodies were in 1:2000 dilutions. Cytochrome C (ab133504), E‐cadherin (20874‐1‐AP) (ab2324), and β‐actin (66009‐1‐Ig) antibodies were in 1:4000 dilutions. The horseradish‐peroxidase labeled goat anti‐rabbit (SA00001‐2) or goat anti‐mouse (SA00001‐1) antibodies were 1:5000 dilution as the secondary antibody. The visualization of protein bands was performed by ECL western blotting substrate (PE0010, Solarbio, Shanghai, China), and the intensities of protein bands were quantified by Image J software (ImageJ 1.51j8, National Institutes of Health, USA).

### 2.4 The detection of PKH26‐labeled MSC‐Exo, HCC‐Exo, and CRC‐Exo for SW1116 cells

PKH26 lipophilic dyes are highly fluorescent and stain membranes by intercalating their aliphatic portion into the exposed lipid bilayer.^34^ Moreover, PKH26 dyes were extensively applied to label and observe the distribution of the exosomes.^35^, ^36^ To observe the exosome uptake for SW1116 cells, MSC‐Exo, HCC‐Exo, and CRC‐Exo were labeled with PKH26 by PKH26 Red Fluorescent Cell Linker Mini Kit (MINI26, Sigma, MO, USA) and were co‐cultured with SW1116 cells. SW1116 cells were incubated with Hoechst 33342 staining solution (C1029, Beyotime Biotechnology, Shanghai, China) at 37°C to obverse the nuclei. Images were captured by a confocal microscope (Leica, Wetzlar, Germany).

### 2.5 Patients and clinical samples

Ten CRC tissue samples and 10 corresponding paracancerous tissues were obtained from patients with CRC in the Department of Oncology and stored immediately in liquid nitrogen after surgery. The Ethical Committee of the First Affiliated Hospital of Harbin Medical University approved this study.

### 2.6 The extraction of RNA and qRT‐PCR

The total RNA extractions of CRC tissue samples and SW1116 cells with different treatments were performed by TsingZol Total RNA Extraction Reagent (TSP401, Tsingke, Nanjing, China). Total RNA (1 μg) was reverse transcribed to cDNA according to the protocol of miRNA first Strand cDNA Synthesis Kit (by stem‐loop) (MR101‐01, Vazyme Biotech Co., Ltd, Nanjing, China). The miRNA Universal SYBR qPCR Master Mix (MQ101‐01/02, Vazyme Biotech Co., Ltd, Nanjing, China) was used to detect the expression of miR‐370‐5p, miR‐1270, and miR‐140‐5p normalized by the housekeeping gene U6. The primers of U6, miR‐370‐5p, miR‐1270, and miR‐140‐5p are displayed in Table 1.

**TABLE 1:** Primers were used for RT‐qPCR.

	Forward (5′‐3′)	Reverse (5′‐3′)
miR‐370‐5p	GCGCAGGTCACGTCTCTGC	AGTGCAGGGTCCGAGGTATT
miR‐1270	CGCGCTGGAGATATGGAAGAG	AGTGCAGGGTCCGAGGTATT
miR‐140‐5p	CGCGCAGTGGTTTTACCCTA	AGTGCAGGGTCCGAGGTATT
U6	CTCGCTTCGGCAGCACATATA	AACGCTTCACGAATTTGCGT

### 2.7 CCK‐8 assay

SW1116 cells were seeded into 96‐well plates (5 × 10^3^ cells/well) and treated with CRC exosomes with miR‐1270, miR‐370‐5p, or miR‐140‐5p overexpression. The cell viabilities were detected by Enhanced Cell Counting Kit‐8 (C0041, Beyotime Biotechnology, Shanghai, China). At several points (0/24/48/72 h), each well was added with the enhanced CCK‐8 solution and incubated for another 2 h. The viabilities for SW1116 cells in all groups were measured at 450 nm absorbance through Multiskan SkyHigh (Thermo Fisher Scientific, MA, USA).

### 2.8 EdU assay

SW1116 cells were treated with CRC exosomes with miR‐1270, miR‐370‐5p, or miR‐140‐5p overexpression, and BeyoClick™ EdU Cell Proliferation Kit assessed the proliferation abilities with Alexa Fluor 488 (C0071S, Beyotime Biotechnology, Shanghai, China). After treating CRC exosomes with different miRNAs overexpression, SW1116 cells with 60% confluence were incubated with Leibovitz's L‐15 medium containing 20 μM EdU reagents for 2 h at 37°C. After removing the medium, SW1116 cells were fixed with 4% paraformaldehyde fix solution (P0099), washed three times with Immunol Staining Blocking Buffer (P0102), and permeabilized by Enhanced Immunostaining Permeabilization Buffer (P0097). All solutions and buffers were purchased from Beyotime Biotechnology (Shanghai, China). In addition, the nuclei were stained with 1X Hoechst 33342 (C0071L‐6, Beyotime, Shanghai, China). EdU‐stained SW1116 cells in all groups were observed and assessed the proliferation ability was assessed by EVOS M5000 fluorescence microscope (Thermo Fisher Scientific, MA, USA).

### 2.9 Cell apoptosis analysis

The apoptosis rates were measured through Annexin V‐FITC/PI Apoptosis Detection Kit (A211‐01, Vazyme Biotech Co., Ltd, Nanjing, China). First, SW1116 cells (2 × 10^5^) in the different groups were collected, washed with pre‐cooled PBS, suspended in 100 μL IX binding buffer, and stained with 6 μL Annexin V‐FITC/propidium iodide (PI) in a dark environment for 25 min incubation. Then, the stained SW1116 cells were suspended by 400 μL IX binding buffer, detected and analyzed by CytoFLEX V2‐B4‐R2 Flow Cytometer (eight detectors, three lasers) (Beckman colter, MA, USA) in 1 h.

### 2.10 Wound‐healing assay

The migration abilities of SW1116 cells in different groups were assessed by wound‐healing assay. SW1116 cells were seeded to 6‐well plates, cultured with Leibovitz's L‐15 medium, and treated with CRC exosomes with miR‐1270, miR‐370‐5p, or miR‐140‐5p overexpression. There was a linear wound by 200 μL pipette when the confluence of SW1116 cells reached 60%–70%. Then, SW1116 cells in all groups were cultured for another 48 h and observed under a CX41 light microscope (Olympus, Tokyo, Japan). Image J software (ImageJ 1.51j8, National Institutes of Health, USA) was used to measure the area of cell migration in five random microscope fields at 0 and 48 h. The wound healing rate under different treatment methods was calculated. Image J software (ImageJ 1.51j8, National Institutes of Health, USA) was used to measure the area of cell migration in five random microscope fields at 0 and 48 h. The wound healing rate under different treatment methods was calculated. Wound healing rate = (wound area at a certain time point/initial wound area)*100%.

### 2.11 Transwell assay

The migration abilities of SW1116 cells in different groups were also implemented by transwell assay. Briefly, SW1116 cells at 6 × 10^4^ cells/mL density, which were incubated with CRC exosomes with miR‐1270, miR‐370‐5p, or miR‐140‐5p overexpression for 24 h, were re‐suspended with 200 μL L‐15 medium (serum‐free) and seeded into the upper chamber, while the lower chamber had 600 μL Leibovitz's L‐15 medium with 20% serum. After the culture for 24 h, non‐invaded SW1116 cells were wiped off, and the upper chamber was fixed with a 4% paraformaldehyde fix solution. Finally, the invaded SW1116 cells were dyed with crystal violet staining solution (C0121, Beyotime, Shanghai, China), captured, and counted under a CX41 light microscope (Olympus, Tokyo, Japan).

### 2.12 Animal studies for the distribution of MSC‐Exo, HCC‐Exo, and CRC‐Exo in vivo

BALB/c nude mice (age, 4–6 weeks) were bought from Cavens Biogel (Suzhou, China), and raised in a clean animal cabinet at 26°C, 12 h light/12 h dark period free to obtain water and complete nutrition food. The mice were injected with 100 μg MSC‐Exo, HCC‐Exo, or CRC‐Exo via the tail vein when the subcutaneous tumor volume of SW1116 cells reached 200 mm^3^. At several points (0.5/3/6 h), the distribution of MSC‐Exo, HCC‐Exo, and CRC‐Exo labeled with PKH26 was monitored and imaged using Xenogen IVIS 100 system (Caliper Life Sciences, MA, USA). After 24 h, mice were euthanized using large doses of intraperitoneal pentobarbital (200 mg/kg). Furthermore, the liver, spleen, lung, kidney, and tumor tissues in MSC‐Exo, HCC‐Exo, and CRC‐Exo groups were separated and captured by Xenogen IVIS 100 system (Caliper Life Sciences, MA, USA). The liver, spleen, lung, kidney, and tumor tissues in MSC‐Exo, HCC‐Exo, and CRC‐Exo groups prepared the frozen section. Concretely, the nuclei were stained with 1X Hoechst 33342 (C0071L‐6, Beyotime, Shanghai, China). The distribution of PKH26‐labeled MSC‐Exo, HCC‐Exo, and CRC‐Exo was observed by EVOS M5000 fluorescence microscope (Thermo Fisher Scientific, MA, USA).

In the experiment of detecting the dominant source of exosomes, the mice were divided into three groups: MSC‐Exo, HCC‐Exo, and CRC‐Exo. In the experiment of detecting the utility of exosomes loaded with miRNA, mice were divided into three groups: CRC‐miR‐1270, CRC‐miR‐370‐5p, and CRC‐miR‐140‐5p. Three mice per group were used as parallel controls, and a total of 18 mice were present for the experimental data.

### 2.13 Tumorigenicity assay in mice

SW1116 cells (5 × 10^6^) were subcutaneously injected into the left axilla of the BALB/c nude mice to generate tumors in mice. After 3 days, CRC exosomes with miR‐1270, miR‐370‐5p, or miR‐140‐5p overexpression (100 μg) were injected into the mice via the tail vein every 3 days six times. The subcutaneous tumor volume analysis was performed with a vernier caliper every 7 days. At the end of the experiment, the mice were euthanized with large doses of intraperitoneal pentobarbital (200 mg/kg), and the tumors were removed for further weight analysis. Moreover, a relative rate of dying for mice was analyzed. All animal experiments were approved by the Ethical Committee of the First Affiliated Hospital of Harbin Medical University (Harbin, China).

### 2.14 Histopathology

To observe pathological changes and the distribution of PKH26‐labeled exosomes in all tissues, the liver, spleen, lung, kidney, and heart tissue samples were frozen in optimum cutting temperature compound embedding media and cryo‐sectioned into 6 μm thick sections by Tissue‐Tek II cryostat. The nuclei were stained with Hoechst 33342 staining solution (C1029, Beyotime Biotechnology, Shanghai, China). Representative images were obtained by a confocal microscope (Leica, Wetzlar, Germany). For the mice of control, Exo‐NC and Exo‐miR‐1270 mimic group, the liver, spleen, lung, kidney, and heart tissues were fixed by tissue fixative for 24 h, dehydrated by dehydrating, wax, embedded, and cut into 10 μm slices. For hematoxylin & eosin (HE) staining, the sections were dewaxed and stained the nucleus after incubating with hematoxylin for 15 min. 1% eosin solution was used to show the symptoms of liver, spleen, lung, kidney, and heart tissues in each group. The graded alcohol could efficiently dehydrate all sections. Representative images were obtained by a light microscope (Olympus, Tokyo, Japan).

### 2.15 Analysis of biochemical indexes for blood

Blood samples were collected and detected the levels of aspartate aminotransferase (AST), alanine aminotransferase (ALT), blood urea nitrogen (BUN), white blood cell (WBC), total bilirubin (T‐Bil), and creatinine (CRE) by FUJI DRI‐CHEM NX500 Series (Fujifilm, Japan).

### 2.16 Statistical analysis

Statistical analysis was performed using GraphPad Prism 7.0 software. Data were demonstrated as means ± standard deviation (SD). In addition, two‐tailed Student's *t*‐test and one‐way ANOVA with Turkey's test was performed to compare the difference between two or multiple groups, respectively. Data with *p* values smaller than 0.05 was considered a significant difference.

## 3 RESULTS

### 3.1 The identification of engineered exosome

**FIGURE 1:**
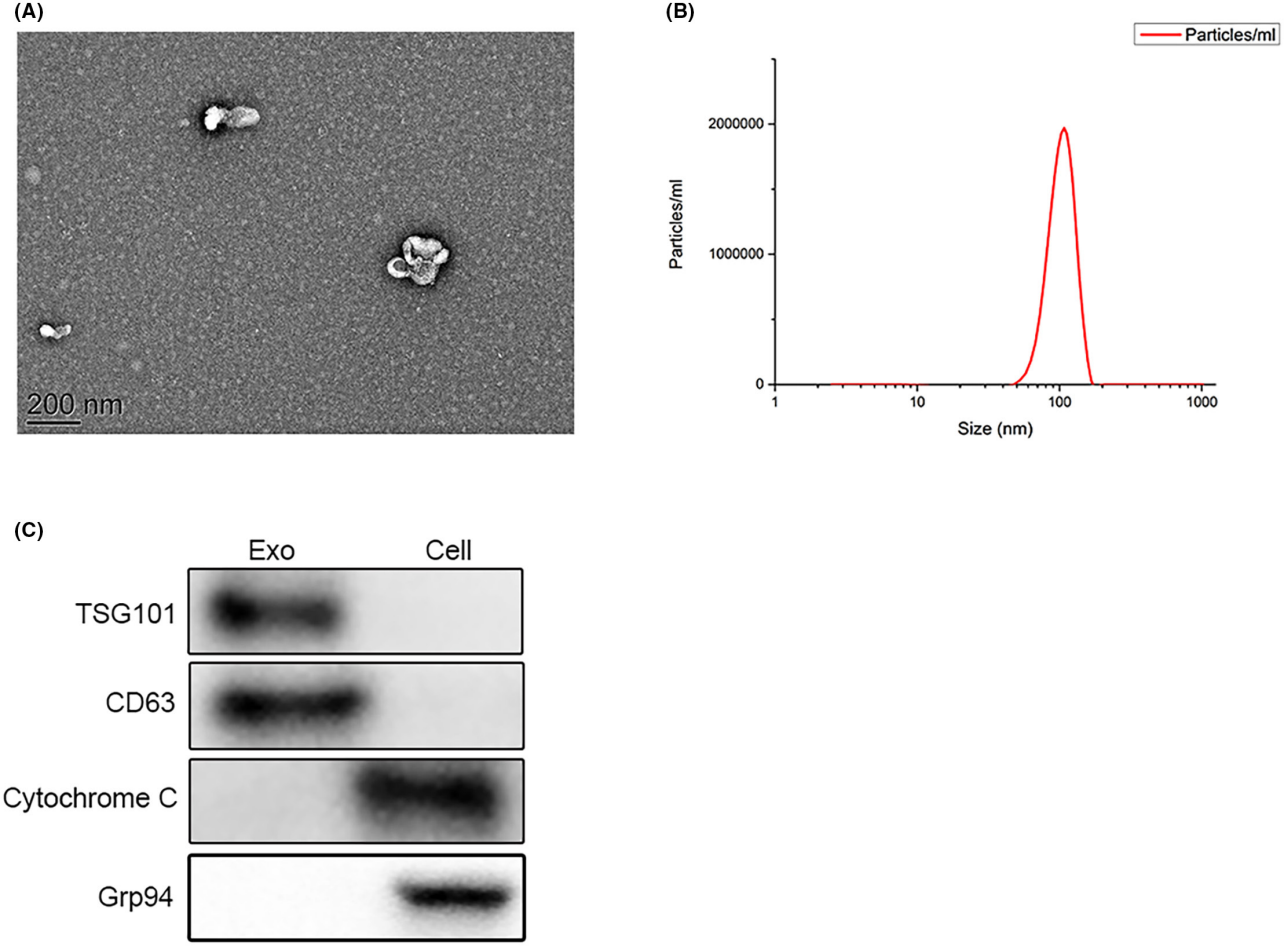
The engineered exosomes were prepared and identified. (A) The shape and structure of engineered exosomes were shown by TEM (scale bar: 200 nm). (B) NTA tracked the diameter of engineered exosomes. (C) The protein expressions of TSG101, CD63, and cytochrome C were detected in engineered exosomes and corresponding cells.

First, in Figure 1A,B, the prepared engineered exosomes were identified by TEM and nanoparticle tracking analysis (NTA), indicating that the engineered exosomes had a 100 nm average diameter and typical two‐layer membrane structure. In Figure 1C, TSG101, and CD63, the representative exosomal markers significantly enriched the Exo group when the corresponding cells were in the control group. As the negative markers, the cytochrome C, and Grp94 proteins were enriched in cells but were absent in exosomes, which suggested that engineered exosomes were successfully prepared.

### 3.2 Specific uptake of engineered CRC exosomes by CRC cells

**FIGURE 2:**
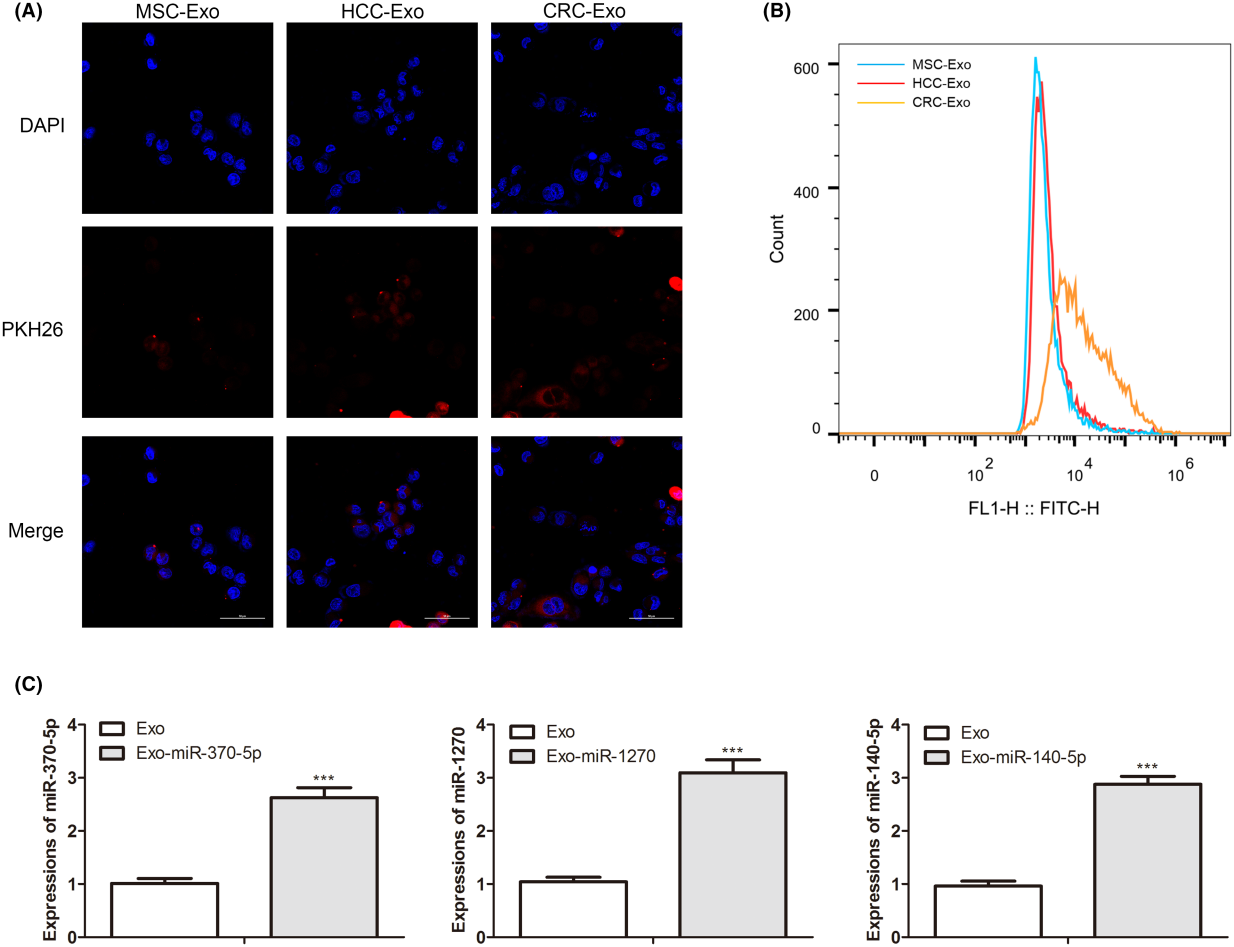
SW1116 cells specifically uptake the engineered CRC exosomes. (A) The green fluorescence levels were detected for SW1116 cells, co‐cultured with PKH26‐labeled MSC‐Exo, HCC‐Exo, and CRC‐Exo (scale bar: 50 μm). (B) Flow cytometry analysis was performed to compare the expression intensity of CD63, a specific marker for the exosomes, in SW1116 cells treated with MSC‐Exo, HCC‐Exo, and CRC‐Exo. (C) The uptake efficiencies of CRC‐Exo for SW1116 cells were assessed by the expression examination of miR‐370‐5p, miR‐1270, and miR‐140‐5p by qRT‐PCR. The * (***p < 0.001) was indicated as the difference with the Exo group.

The prepared MSC‐Exo, HCC‐Exo, and CRC‐Exo were labeled with PKH26. In Figure 2A, PKH26‐labeled MSC‐Exo, HCC‐Exo, and CRC‐Exo were co‐cultured with SW1116 cells for 3 h, and there was the most robust red fluorescence in the CRC‐Exo group compared with MSC‐Exo and HCC‐Exo group. In Figure 2B, flow cytometry analysis showed that the highest level of CD63 appeared in the CRC‐Exo group. These results suggested that CRC‐Exo could be specifically uptaken by SW1116 cells. It was reported that the engineer exosomes effectively load and deliver exogenous nucleic acids such as miRNA to cancer cells.^27^ Therefore, we wanted to design the functional CRC‐Exo to deliver the carcinostatic miRNA to regulate the development of CRC. Wang et al. proved that miR‐140‐5p partly inhibited the effect of lncRNA cancer susceptibility 19 from exerting the tumor suppression pattern in CRC.^37^ More and more evidence revealed that miR‐1270 had a tremendous therapeutic significance because miR‐1270 could inhibit the progression of various cancers such as human glioblastoma cancer, cervical cancer, and ovarian cancer.^10^, ^38^, ^39^ Moreover, in our previous studies, miR‐370‐5p, miR‐1270, and miR‐140‐5p were the targets for LncRNA CASC19 in CRC. Here, we tested the expression levels of miR‐370‐5p, miR‐1270, and miR‐140‐5p in CRC tissues and cell lines by qRT‐PCR analysis. MiR‐1270 and miR‐140‐5p had low expression in the human CRC tissue samples, SW1116 cells, and SW480 cells, compared with non‐tumor tissue samples and healthy colon epithelial cells in Figure S1A,B (*p* < 0.001). Moreover, the expression level of miR‐370‐5p had no significant changes. MiR‐370‐5p, miR‐1270, and miR‐140‐5p were loaded into the exosomes of CRC cells by electroporation. Loading efficiency of different miRNAs into CRC exosomes was estimated by qRT‐PCR analysis. In Figure S2, high levels of miR‐370‐5p, miR‐1270, and miR‐140‐5p appeared in the Exo‐miR‐370‐5p, Exo‐miR‐1270, and Exo‐miR‐140‐5p group (*p* < 0.0001), indicating that CRC exosomes had a high loading efficiency for multiple miRNAs. MiR‐370‐5p/miR‐1270/miR‐140‐5p‐loaded CRC exosomes were incubated with SW1116 cells for 24 h, and the uptake efficiency was assessed. QRT‐PCR analysis in Figure 2C showed that the relative expressions of miR‐370‐5p, miR‐1270, and miR‐140‐5p were significantly upregulated in the miR‐370‐5p/miR‐1270/miR‐140‐5p‐loaded exosomes group (*p* < 0.0005), clarifying that miRNAs‐loaded exosomes from CRC cells could be uptaken explicitly by SW1116 cells and deliver exogenous miRNAs to SW1116 cells.

### 3.3 Engineered CRC exosomes loaded with miR‐1270 effectively inhibited the proliferation and promoted apoptosis in CRC cells

**FIGURE 3:**
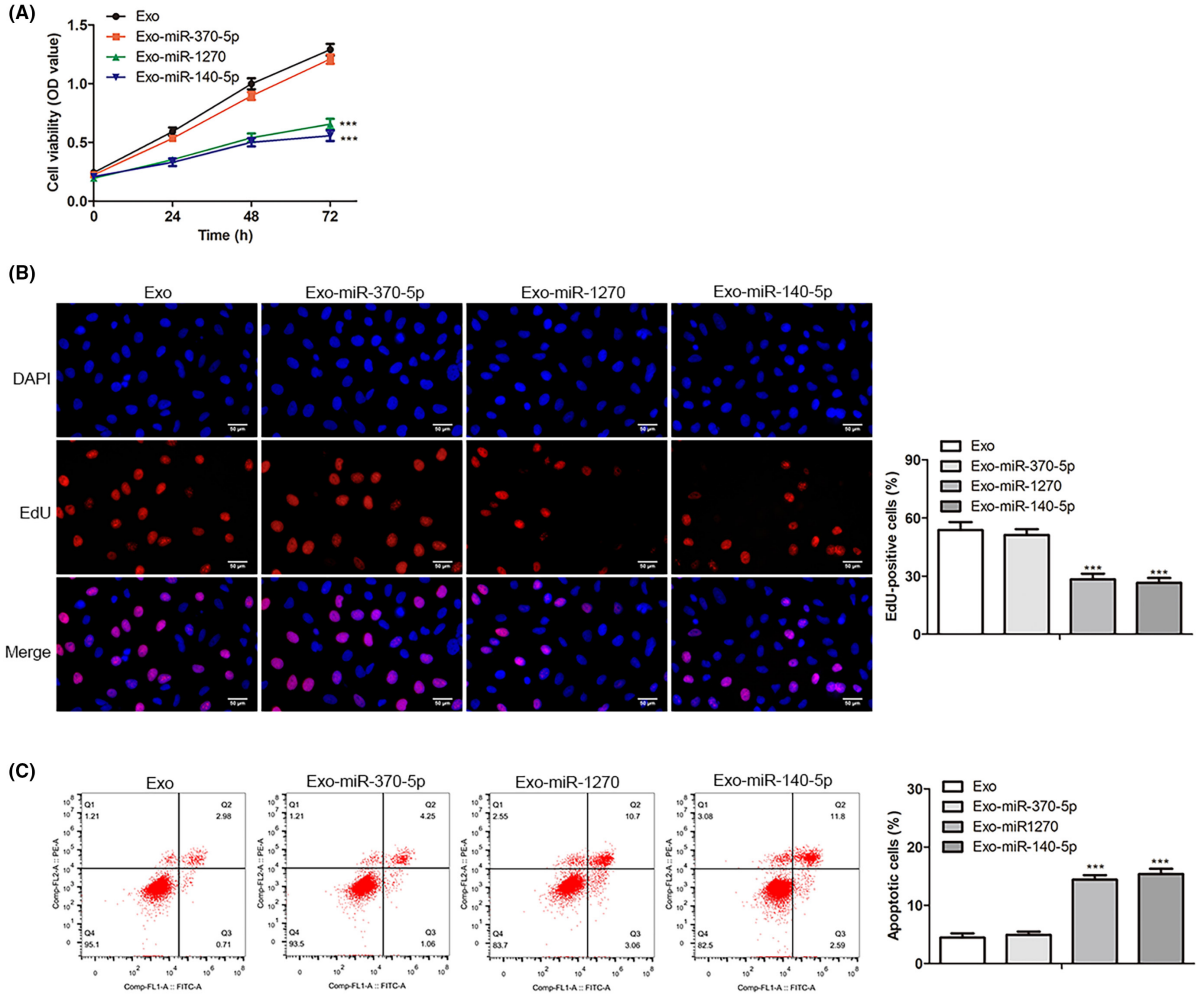
The proliferation ability and apoptosis rate alteration of SW1116 cells affected by CRC exosomes with miR‐1270, miR‐370‐5p, or miR‐140‐5p overexpression. (A) After the treatment of CRC exosomes with miR‐1270, miR‐370‐5p, or miR‐140‐5p overexpression, CCK‐8 assays tested the proliferation ability of SW1116 cells (0, 24, 48, and 72 h). (B) The proliferation abilities of SW1116 cells, which were incubated with Exo‐miR‐1270, Exo‐miR‐370‐5p, or Exo‐miR‐140‐5p, were checked by EdU assay (scale bar: 50 μm). (C) Flow cytometry analyzed the apoptosis rate in all groups. The * (***p < 0.001) was indicated as the difference with the Exo group.

To determine the effect of engineered CRC exosomes on the proliferation of SW1116 cells, CCK8, and EdU assays were performed. In Figure 3A, relative to the Exo group (the engineered CRC exosomes without miRNA), CRC exosomes loaded with miR‐1270 or miR‐140‐5p presented a significant proliferation inhibition (*p* < 0.0001). However, there was no apparent difference in the OD value (450 nm) in the Exo‐miR‐370‐5p group. In Figure 3B, a relatively lower percentage of EdU^+^ proliferating SW1116 cells was observed in the Exo‐miR‐1270 and Exo‐miR‐140‐5p groups compared with the Exo group (*p* < 0.0001). Flow cytometry assay in Figure 3C showed that SW1116 cells co‐cultured with Exo‐miR‐1270 and Exo‐miR‐140‐5p had a high apoptosis rate in comparison to the Exo group (*p* < 0.0001). In contrast, its apoptosis ratio had no changes for the Exo‐miR‐370‐5p group, which suggested that CRC exosomes loaded with miR‐1270 excessively inhibited the proliferation and enhanced the apoptosis for SW1116 cells.

### 3.4 Engineered CRC exosomes loaded with miR‐1270 effectively inhibited the migration of CRC cells

**FIGURE 4:**
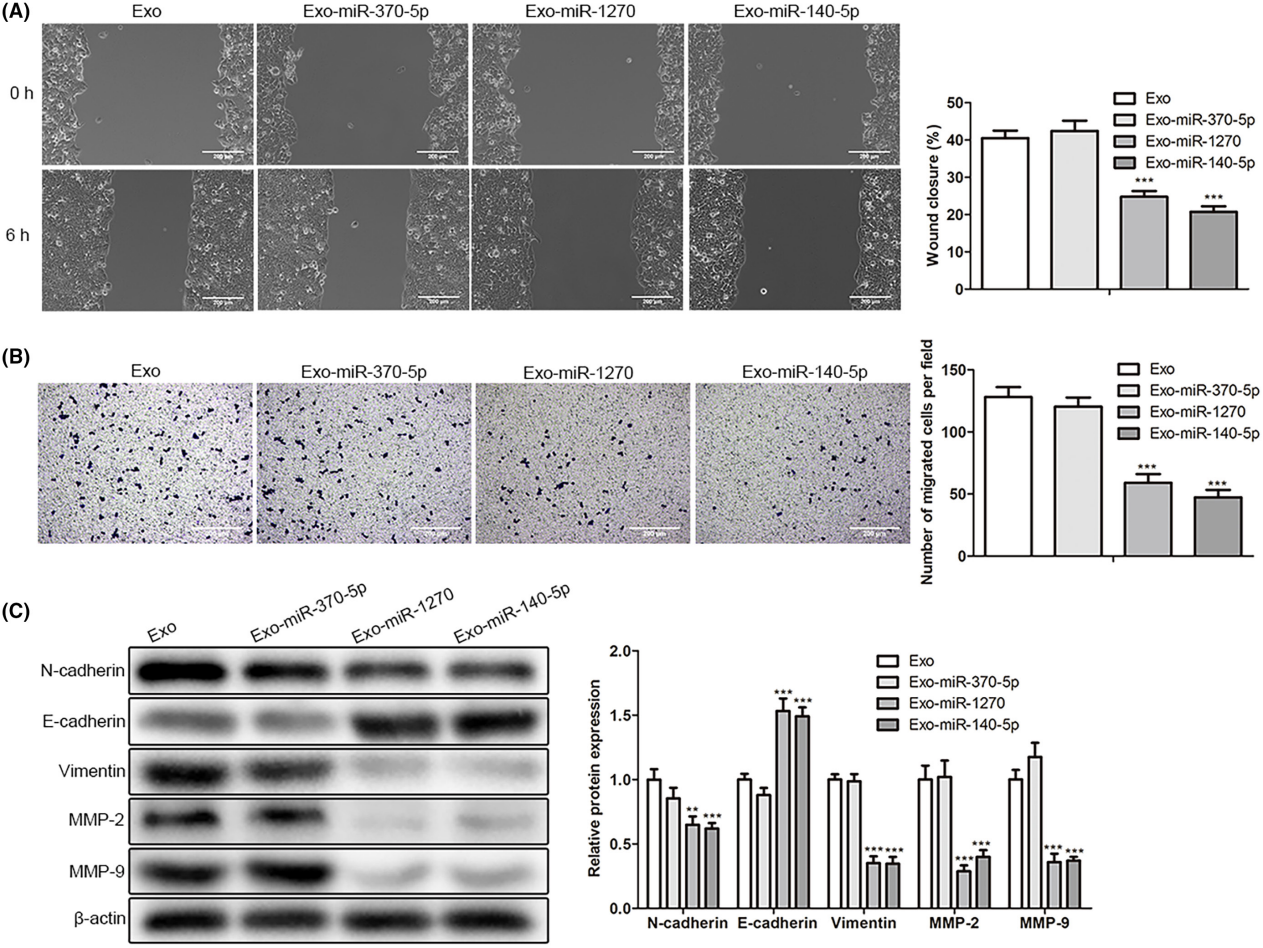
The migration ability and invasiveness changes of SW1116 cells affected by CRC exosomes loaded with miR‐1270, miR‐370‐5p or miR‐140‐5p. (A) Scratch assay was used to analyze the migration abilities of SW1116 cells in Exo, Exo‐miR‐370‐5p, Exo‐miR‐1270, and Exo‐miR‐140‐5p groups (scale bar: 200 μm). (B) After the incubation of CRC exosomes loaded with miR‐1270, miR‐370‐5p, or miR‐140‐5p, the invasiveness of SW1116 cells was examined (scale bar: 200 μm). (C) In all groups, the migration and EMT‐related protein expression levels (MMP‐2/9, N‐cadherin, N‐cadherin, and vimentin) were estimated by western blot. The * (**p < 0.05; ***p < 0.01) was indicated as the difference with the Exo group.

Scratch and transwell test results showed that Exo‐miR‐1270 and Exo‐miR‐140‐5p attenuated the migration and invasion abilities of SW1116 cells in Figure 4A,B. In addition, in Figure 4C, western blot analysis showed that the migration‐related proteins such as matrix metalloproteinase (MMP‐2/9) and EMT‐related proteins (N‐cadherin and vimentin) were downregulated. In SW1116 cells, the E‐cadherin expression level was upregulated after treating CRC exosomes with miR‐1270 or miR‐140‐5p overexpression, not miR‐370‐5p. All results illustrated that CRC exosomes loaded with miR‐1270 effectively inhibited migration abilities for CRC cells (*p* < 0.0001).

### 3.5 Engineered CRC exosomes are mainly distributed in tumors

**FIGURE 5:**
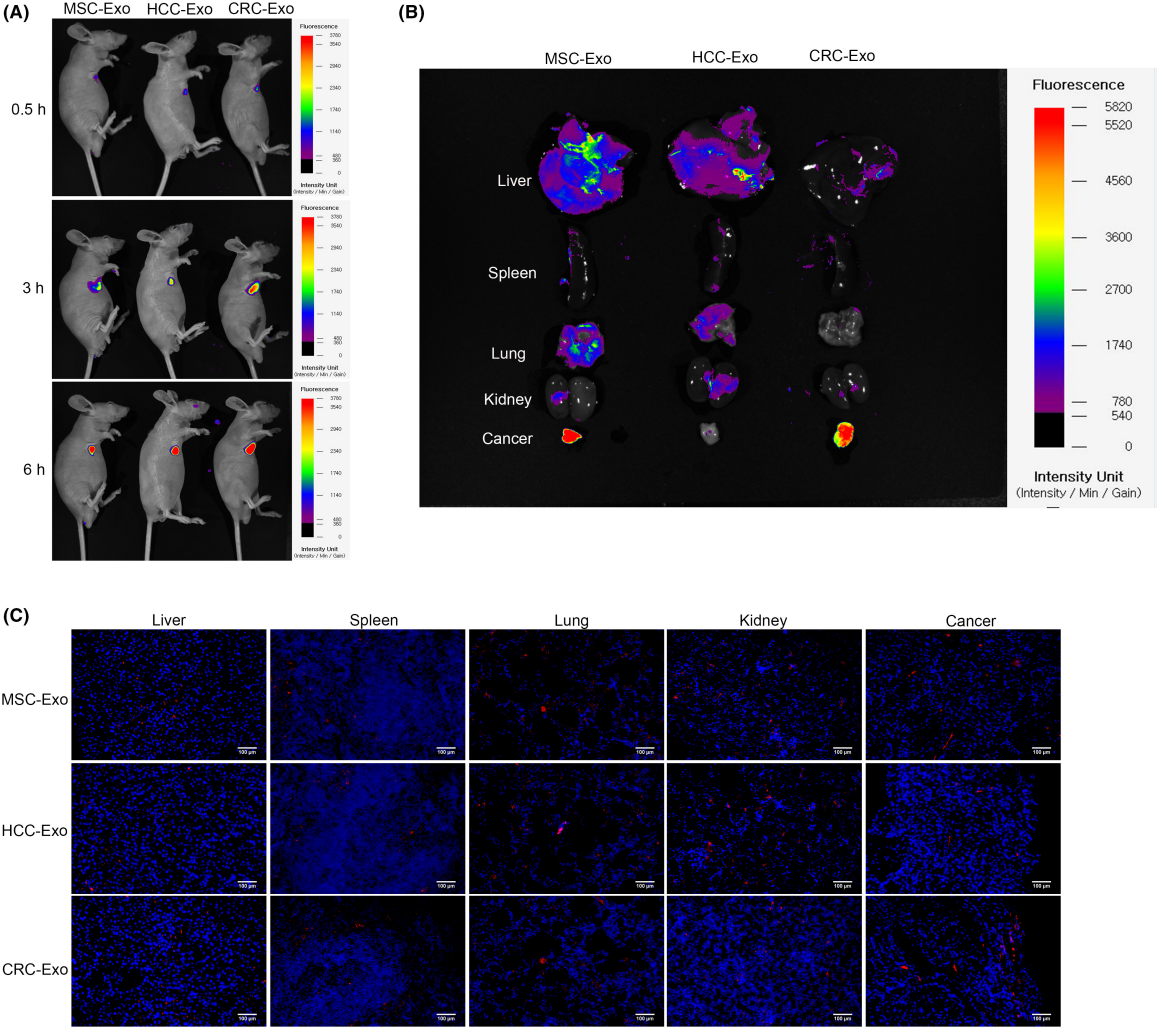
Distribution of engineered exosomes in vivo. (A) After injecting the PKH26‐labeled MSC‐Exo, HCC‐Exo, and CRC‐Exo, the fluorescence distribution in mice was examined when the tumor volume reached approximately 200 mm^3^. (B) MSC‐Exo, HCC‐Exo, and CRC‐Exo‐mediated delivery in vivo for mice were observed, including the liver, spleen, lung, kidney, and tumor. (C) The fluorescence intensities of the liver, spleen, lung, kidney, and tumor tissue sections were observed after treating MSC‐Exo, HCC‐Exo, and CRC‐Exo (scale bar: 100 μm).

MSC‐Exo, HCC‐Exo, and CRC‐Exo‐mediated delivery in vivo had been evaluated. The immunodeficient nude mice were injected with SW1116 cells. Specifically, MSC‐Exo, HCC‐Exo, and CRC‐Exo were labeled with PKH26, injected into the nude mice bearing approximately 200 mm^3^ tumors, and monitored at 0.5, 3, and 6 h by Xenogen IVIS 100 system. In Figure 5A, there was abundant fluorescence distribution in the mice injected with CRC‐Exo at 3 and 6 h. In addition, In Figure 5B, after treating MSC‐Exo, HCC‐Exo, and CRC‐Exo labeled with PKH26 for 48 h, fluorescence images of the liver, spleen, lung, kidney, and tumor in all groups were obtained and captured under Xenogen IVIS 100 system. MSC‐Exo was accumulated in cancer tissue like CRC‐Exo. In MSC‐Exo, HCC‐Exo, and CRC‐Exo groups, the liver, spleen, lung, kidney, and tumor tissues were collected, sectioned, stained, and imaged. In Figure 5C, a significant red fluorescence signal was observed in the CRC‐Exo group, especially tumor tissue.

### 3.6 CRC exosomes loaded with miR‐1270, miR‐370‐5p, or miR‐140‐5p affected the tumor growth and overall survival rate for mice in vivo

**FIGURE 6:**
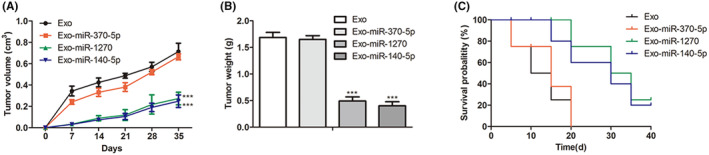
In vivo efficacy evaluation for CRC exosomes loaded with miR‐1270, miR‐370‐5p or miR‐140‐5p. (A) The tumor volumes in all groups were measured every 7 days. (B) The tumor weights in all groups were measured when xenograft mice were sacrificed. (C) Overall survival rates were counted in different groups. The * (***p < 0.001) was indicated as the difference with the Exo group.

To inquire about the effect of CRC exosomes loaded with miR‐1270, miR‐370‐5p, or miR‐140‐5p on the tumor growth potential in vivo, the immunodeficient nude mice were injected with SW1116 cells. After 7 days, the mice were treated with Exo, Exo‐miR‐370‐5p, Exo‐miR‐1270, or Exo‐miR‐140‐5p, respectively, every 3 days for seven times. After 28 days postinjection, the Exo, Exo‐miR‐370‐5p, Exo‐miR‐1270, and Exo‐miR‐140‐5p groups measured tumor weight and size. The maximum tumor volume was 0.800 cm^3^ (Exo), 0.695 cm^3^ (Exo‐miR‐370‐5p), 0.340 cm^3^ (Exo‐miR‐1270), and 0.315 cm^3^ (Exo‐miR‐140‐5p) when the mice were sacrificed. In Figure 6A, miR‐1270 or miR‐140‐5p overexpression in vivo significantly inhibited tumor growth for CRC (*p* < 0.0001). In Figure 6B, the tumor weights in the Exo‐miR‐1270 and Exo‐miR‐140‐5p groups were remarkably reduced than the Exo group (*p* < 0.0001). Consistently, in Figure 6C, the mice in the Exo‐miR‐1270 and Exo‐miR‐140‐5p group showed a relatively decreased risk of dying compared to those in the Exo and Exo‐miR‐370‐5p group. Thus, it indicated that Exo‐miR‐1270 played a tumor‐suppressive role in CRC development in vivo.

### 3.7 CRC exosomes loaded with miR‐1270 had no toxicity for other organs of mice in vivo

**FIGURE 7:**
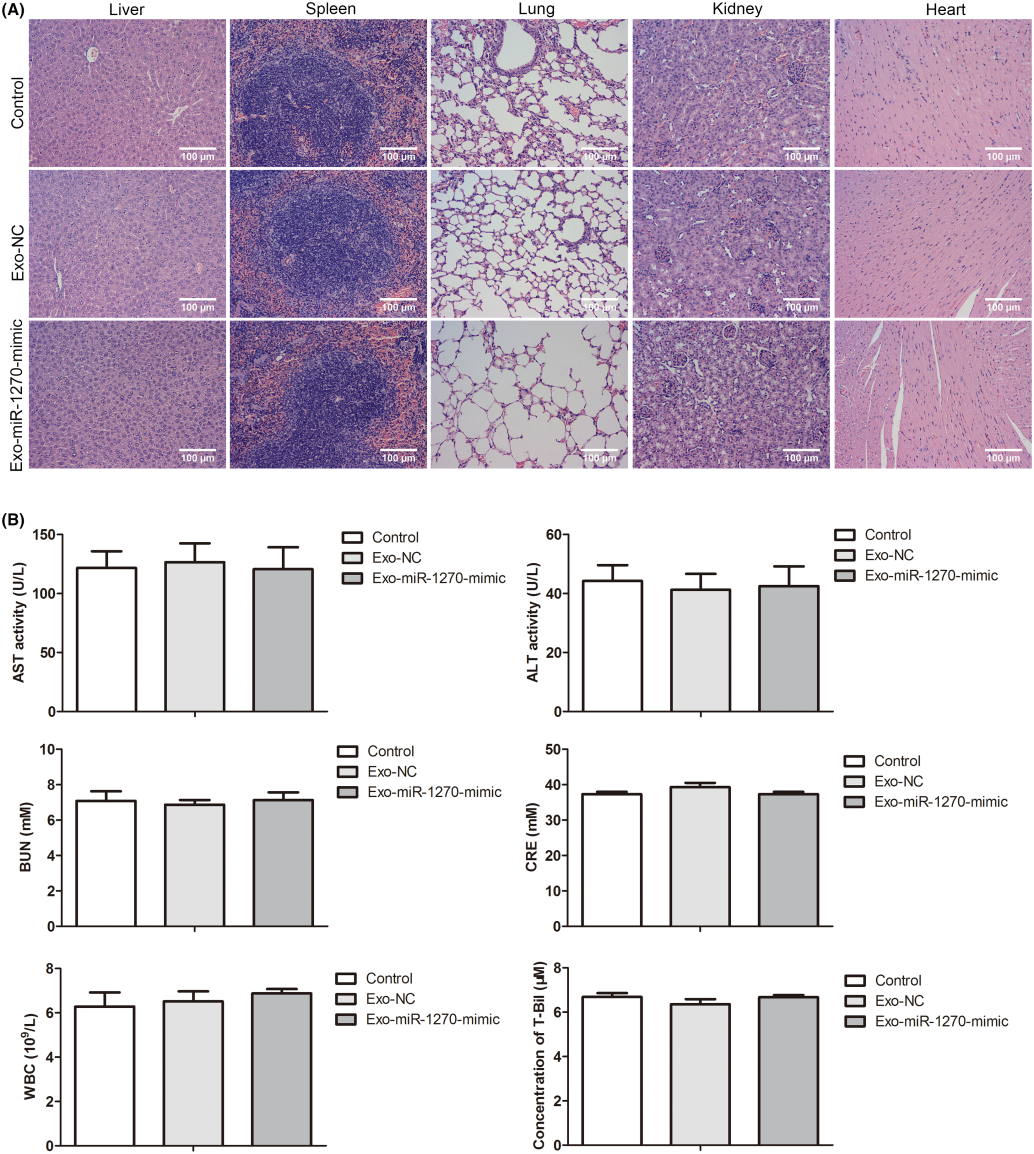
The safety evaluation for CRC exosomes loaded with miR‐1270 in vivo. (A) HE staining results in the control, Exo‐NC, and Exo‐miR‐1270 groups were shown for the liver, spleen, lung, kidney, and heart tissues (scale bar: 100 μm). (B) The chemistry and hematology parameters in mice were tested in all groups.

Besides the efficacy evaluation, toxicity was also a vital parameter of CRC exosomes loaded with miR‐1270 in vivo. H&E staining results showed that the liver, spleen, lung, kidney, and heart tissues in the Exo‐miR‐1270 group were not remarkable changes relative to the control or Exo‐NC group (Figure 7A). The biochemistry parameters were tested, including the markers for liver function (ALT and AST), the biomarkers for kidney function (CRE and blood BUN), and the index for hematological assessment (T‐Bil and WBC). Furthermore, these indexes had no significant changes for comparing the control, Exo‐NC, and Exo‐miR‐1270 group (Figure 7B). Thus, CRC exosomes loaded with miR‐1270 overexpression had no toxicity for the major organs and did not influence the hematological parameters in mice (*p* > 0.05).

## 4 DISCUSSION

Engineered exosomes could exist stably and had low immunogenicity in blood to cross the biological barriers to mediate various complex biological processes and involve in the various cancer progression.^40^, ^41^, ^42^, ^43^ In addition, engineered exosomes attracted much attention because they effectively delivered exogenous biomolecules, such as nucleic acids and peptides, or therapeutic drugs to targeted cells. Moreover, engineered exosomes had an excellent therapeutic effect in the treatment of various cancers and diseases. For instance, Gomari introduced a particular type of engineered and targeted exosomes. Moreover, these exosomes loaded and delivered the doxorubicin to HER2+ cancer cells to elevate anticancer effects.^21^ In colon cancer, it was reported that engineered exosomes could co‐deliver miR‐21 inhibitor oligonucleotide to downregulate the miR‐21 expression and 5‐Fluorouracil to reverse 5‐FU‐resistance for chemotherapeutics.^44^ The engineered exosomes efficiently delivered the therapeutic miR‐26a to HepG2 cells to suppress the malignant development of hepatocellular carcinoma.^27^ Our work found that miR‐1270 had a low expression in the human CRC tissue samples and cell lines. CRC‐Exo loaded with miR‐1270 overexpression could be effectively absorbed by SW1116 cells and weakened CRC proliferation, migration, and tumor growth. In addition, miR‐1270 overexpression by CRC‐Exo delivery promoted the apoptosis rates and inhibited the migration‐related protein and EMT‐related protein expressions in SW1116 cells. Therefore, it demonstrated that CRC‐Exo loaded with miR‐1270 overexpression served as an anticancer effector molecule in CRC progression.

It is noteworthy that the engineered exosomes from HEK 293 T cells or other nonhomologous cells usually were modified by exosomal membrane proteins to enhance the transfer efficiency of functional macromolecules.^45^ For instance, the mouse immature dendritic cells‐derived exosomes were modified by the exosomal membrane protein lysosome‐associated membrane glycoprotein 2b to deliver the doxorubicin to inhibit tumor growth.^46^ Co‐overexpression of exosomal membrane proteins CD63 and Apo‐A1 improved the delivery efficiency of engineered exosomes derived from 293 T cells.^27^ In this study, we selected the SW1116 cells as the cell source to produce the engineered exosomes for the treatment of CRC because they had a high yield and homology. Moreover, the high homology might be advantageous to exosomes to effectively evade phagocytic clearance from the circulation and target the tumor of CRC.

For the application of engineered exosomes in various diseases and cancers, safety is also an essential indicator beside the benefits of targeted delivery. The engineered exosomes were combined with the vanadium carbide quantum dots photothermal agents to destruct the tumors to achieve low‐temperature photothermal therapy. Moreover, these engineered exosomes had no significant side effects, toxicities, and evident inflammations or damages.^47^ The engineered exosomes with highly efficient delivery of CRISPR‐Cas9 components exhibited little immunogenicity and toxicity.^48^ The indocyanine green‐loaded exosomes, surface‐engineered with an active folic acid, could serve as effective nanosonosensitizers for safe, and targeted cancer treatment characterized without systemic toxicity.^49^ In this study, CRC exosomes loaded with miR‐1270 had no toxicity for significant organs and did not affect mice's chemistry and hematology parameters in vivo. However, some limitations still existed in this research. For example, the target of miR‐1270 was not investigated to inhibit the proliferation of colon cancer; and the SW1116 cell line, which is from human colorectal adenocarcinoma, is not in line with the subject of this study CRC accurately. We will gradually improve these problems in the following research.

Recent studies have found that exosomes act as essential transporters of various non‐coding RNAs (ncRNAs), through which they mediate almost all cancer markers leading to CRC metastasis. Among them, both circular RNA (circrna) and long non‐coding RNA (lncrna) mostly act as a sponge for miRNA to further regulate CRC. For example, exosomal circ‐133 promotes CRC metastasis by regulating miRNA‐133a,^50^ and exosomal circFNDC3B inhibits CRC angiogenesis and metastasis by negatively targeting miRNA‐937‐5p.^51^ Exosomal MALAT1 promotes the malignant metastasis of CRC cells by adsorbing miRNA‐26a/26b^52^ et al. And, Recent reports have shown that mirNA‐335‐5p, miRNA‐135b‐5p, miRNA‐934, miRNA‐106b, miRNA‐27b‐3p, mirNA‐106b‐3p, miRNA‐221/222 and miRNA‐203 are all upregulated in CRC tissue cells Promotes the transfer of a cell and the attack, but the miRNA‐3940‐5 p, micrornas‐16‐5 p, the miRNA‐424, the miRNA‐140‐3‐p in CRC expression by means of tissue cells are inhibits cell metastasis and invasion, promoted the apoptosis.^53^ Among them, there is a lack of studies on miR‐1270, which is supplemented in this paper.

## 5 CONCLUSION

In conclusion, the homologous tumor‐derived exosomes loaded with miR‐1270 in this study might serve as essential tools to efficiently deliver miR‐1270 and selectively enhance the suppression effect for colorectal adenocarcinoma cells in vitro and inhibit the tumor growth of CRC in vivo. Thus, it provided novel therapeutic opportunities for the treatment of CRC.

## AUTHOR CONTRIBUTIONS

**Yingmin Jin:** Conceptualization (lead); formal analysis (lead); funding acquisition (lead); project administration (lead); resources (equal); supervision (lead); validation (lead); writing – original draft (equal); writing – review and editing (lead). **Liying Sun:** Data curation (equal); formal analysis (equal); investigation (equal); methodology (equal); software (equal); supervision (equal); validation (equal); writing – original draft (equal). **Yingying Chen:** Data curation (equal); formal analysis (equal); investigation (equal); methodology (equal); software (equal); validation (equal); visualization (equal). **Yue Lu:** Data curation (equal); investigation (equal); methodology (equal); software (equal); visualization (equal); writing – original draft (equal).

## FUNDING INFORMATION

This research received no specific grant from any funding agency in the public, commercial, or not‐for profit sectors.

## CONFLICT OF INTEREST STATEMENT

The authors declared no potential conflicts of interest with respect to the research, authorship, and/or publication of this article.

## ETHICS STATEMENT

The experimental protocol was established according to the ethical guidelines of the Helsinki Declaration and was approved by the Ethical Committee of the First Affiliated Hospital of Harbin Medical University.

## PATIENT CONSENT STATEMENT

All experiments were performed after obtaining the informed consent signed by the patients.

## Supporting information


Figure S1.

Figure S2.

## Data Availability

All data generated or analyzed during this study are included in this article and its supplementary material files. Further enquiries can be directed to the corresponding author.
