# Supplementary figures and images for: The homologous tumor‐derived‐exosomes loaded with miR‐1270 selectively enhanced the suppression effect for colorectal cancer cells
Source: Cancer Med. 2024 Jan 10;13(1):e6936. doi: 10.1002/cam4.6936 (PMC10807586; doi:10.1002/cam4.6936)

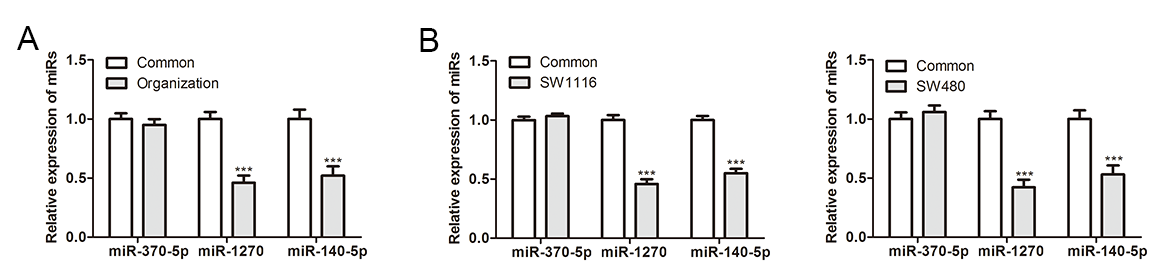

Supplement: Supplementary file 1 — Figure S1. [file CAM4-13-e6936-s001.tif]

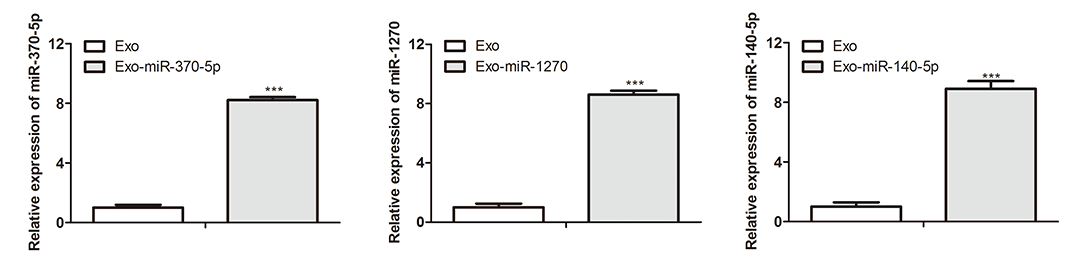

Supplement: Supplementary file 2 — Figure S2. [file CAM4-13-e6936-s002.tif]
